# Human bocavirus (HBoV) in Kuwait: molecular epidemiology and clinical outcome of the virus among patients with respiratory diseases

**DOI:** 10.1099/jmm.0.001219

**Published:** 2020-06-24

**Authors:** Nada M. Madi, Anfal Al-Adwani

**Affiliations:** ^1^​ Virology Unit, Department of Microbiology, Faculty of Medicine, Kuwait University, Kuwait

**Keywords:** bocavirus, epidemiology, Kuwait, respiratory patients

## Abstract

**Introduction:**

Globally, human bocavirus (HBoV) has been detected in respiratory samples from patients suffering from upper and lower respiratory diseases. In Kuwait, little is known about the epidemiological and clinical characterization of the virus and genetic characterization of the virus as a respiratory pathogen is unknown.

**Aim:**

This study aims to explore the molecular epidemiology and clinical features of HBoV isolates in patients with respiratory diseases.

**Methodology:**

Retrospectively, between 2018 and 2020, 5941 respiratory samples from patients with respiratory diseases were screened for respiratory viruses using multiplex real-time PCR. Samples that were positive for HBoV were then subjected to NP1 and VP1/PV2 phylogenetic analysis.

**Results:**

HBoV was detected in 1.9 % of the patients, with a peak incidence of infection among children <1 year old. Co-infection with other respiratory viruses was observed in 56.8 % of HBoV-positive patients. Fever, cough and respiratory distress were the most common clinical features of HBoV infection. Phylogenetic analysis of the Kuwaiti HBoV isolates revealed that all the isolates were of the HBoV-1 genotype, with slight sequence variations among the isolates.

**Conclusion:**

This study illustrated the predominance of the HBoV-1 genotype in patients with respiratory diseases in Kuwait with minimal genetic variability. It also highlighted the clinical features of HBoV-1 infection, verifying its role in respiratory diseases.

## Introduction

Human bocavirus (HBoV) was first revealed in 2005 in respiratory samples from patients with suspected acute respiratory tract infection (ARTI) using metagenomics approaches [1]. HBoV-1 has predominantly been diagnosed in the respiratory tract. However, other related HBoV types, namely HBoV2, HBoV3 and HBoV4, were discovered later and have been found mainly in human stool samples [2]. HBoV is a small non-enveloped single-stranded DNA virus that belongs to the family *Parvoviridea* and the subfamily *Parvovirinae*. The viral DNA genome is approximately 5 Kb in length, and encodes two non-structural proteins, NS1 and nuclear phosphoprotein 1 (NP1), and two major structural proteins, VP1 and VP2 [3–6]. NS1 has regulatory functions, including transactivation or induction of apoptosis [7, 8]. On the other hand, the specific functions of NP1 are so far unknown, while VP1/VP2 are viral capsid proteins [8–10]. NS1 gene has the lowest genetic diversity among all HBoV subtypes; therefore, this gene has been used successfully as a target for HBoV detection [11]. By contrast, VP1/VP2 genes exhibit greater genetic diversity and are used for phylogenetic analysis of HBoV [12].

HBoV is distributed worldwide. It has been detected in many countries, including Australia, the USA, Japan, Sweden, Germany, Jordan, South Africa, France, Canada, Iran, Spain, The Netherlands, the Republic of Korea, Thailand, Switzerland and PR China. The epidemiological data show that the global prevalence of HBoV in acute respiratory infections is 6.3 % [confidence interval (CI): 6.2–6.4], while in gastrointestinal infections it is 5.9 % (CI: 5.7–6.1) [13].

The role of HBoV in respiratory diseases is currently being debated. Numerous studies have shown an association between HBoV-1 and upper and lower respiratory tract diseases. The most common clinical presentations of HBoV include cough, fever, rhinitis, asthma exacerbation, acute wheezing, acute otitis media and pneumonia [1, 13–15]. However, HBoV can also be detected in asymptomatic people [15]. The fact that HBoV is prevalent in samples from patients with ARTI does not assure a causative role of the virus in the disease because many viruses are transmitted via the respiratory tract without causing significant respiratory symptoms. However, verifying the causative role of a specific agent in disease requires multiple studies [16]. HBoV is frequently present alongside other respiratory viruses. Studies reported a high percentage of co-infection, with frequencies of 18–90 % being reported [1, 17].

In Kuwait, the epidemiology, genetic diversity and clinical involvement of HBoV in respiratory diseases are unknown. Thus, the present study aims to evaluate the prevalence and genetic diversity of HBoV in patients with respiratory diseases in Kuwait and to study clinical outcomes among these patients.

## Methods

### Study population and specimen collection

Retrospectively, between January 2018 and January 2020, a total of 5941 respiratory samples were collected from hospitalized patients with respiratory tract infections (RTIs) at different hospitals in Kuwait. Upon physical examination and X-rays by the physicians, various respiratory diseases were recorded in these patients, such as bronchiolitis, pneumonia, acute respiratory distress syndrome (ARDS), croup, bronchopneumonia and acute nasopharyngitis. Nasopharyngeal aspirates/wash, tracheal aspirates and throat swabs were collected from the patients and transported immediately at 4 °C to the Virology Unit, Medical Laboratory, Mubarak Al-Kabeer Hospital, where they were stored at −70 °C until use. The collected samples were first screened for respiratory viruses, including HBoV, using multiplex real-time PCR. HBoV-positive samples were further subjected to molecular analysis by specific bocavirus PCR and phylogenetic analysis after nucleic acid sequencing at the Virology Unit, Faculty of Medicine, Kuwait University. Patients demographics and clinical data, including medical history, signs and symptoms, were all collected.

### Multiplex real-time PCR for respiratory viruses including HBoV

Viral RNA or DNA was extracted from respiratory samples using the Roche MagNA Pure LC system (Roche Diagnostics, Indianapolis, IN, USA), according to the manufacturer’s instructions. Extracted nucleic acids from all samples were tested for HBoV along with other respiratory viruses using the one-step multiplex real-time PCR assay. This assay can detect influenza A, influenza A (H1N1) swl, influenza B, human rhinovirus (HRV), human coronavirus NL63 (HCoV-NL63), HCoV-229E, HCoV-OC43, HCoV-HKU1, parainfluenza (PIV) 1, 2, 3, 4, human metapneumovirus (HMPV) A/B, human bocavirus, respiratory syncytial virus (RSV) A/B, adenovirus (AdV), enterovirus, parechovirus, mycoplasma pneumonia and internal control using the Fast Track kit (Fast Track Diagnostics, Luxembourg). The amplification was performed according to the manufacturer’s instructions.

### Sequencing of NP1, VP1 and VP2 genes of HBoV

For sequence analysis, HBoV-positive samples were screened for the 779 nt fragment at the NP1, VP1 and VP2 gene boundary using primers HBoV-NP1/VP/1 (5′-GGTGGTGTGGGTTCTACTGG-3′) and HBoV-NP1/VP/R (5′-GAGGTGTTTTGTGGTGCGTC-3′). The amplification profile comprised an initial denaturation at 95 °C for 5 min, followed by 35 amplification cycles (94 °C for 1 min, 54 °C for 1 min and 72 °C for 2 min), with a final extension at 72 °C for 10 min. The amplicon was analysed by electrophoresis in a 1.5 % (W/V) agarose gel. The PCR products were purified with a QIAquick gel extraction kit (Qiagen) according to the manufacturer’s instructions. Subsequently, the purified products were sequenced in the forward and the reverse directions by the forward primer HBoV-NP1/VP/1 in addition to the reverse primer HBoV-NP1/VP/R. Sequencing was performed using the ABI 3500/3500xL genetic analyzer (PE Applied Biosystems, Inc., Foster City, CA, USA) with the ABI Prism BigDye Terminator Cycle Sequencing Ready Reaction kit (PE Applied Biosystems, Inc., Foster City, CA, USA).

### Gene sequence analysis

blast searches for amplicon sequences were carried out using sequence similarity against available HBoV sequences in the GenBank database (https://www.ncbi.nlm.nih.gov/). Nucleotide sequences were edited and aligned manually with the available HBoV sequences from the GenBank database using the clustal W method and mega software version 7 [18]. The sequences were presented in a topology tree prepared using mega software version 7. The nucleotide distance matrix was created using the maximum-likelihood method based on the Tamura–Nei substitution model with uniform rates among sites and complete gap deletion. Bootstrap presampling (1000 replications) was used to measure the reliability of individual nodes in the phylogenetic tree. As for comparative phylogenetic analysis, the following HBoV strains sequences published in the National Centre for Biotechnology Information (NCBI) database were used: HBoV-1: KG684067, GQ926982, MG383449, MG953829, KU557404, KX373885, KP710204, KJ684073, MF376168, JX434066, MG953830, JX434082; HBoV-2: FJ170278; HBoV-3: EU918736; HBoV-4: FJ973561).

## Results and Discussion

During the 2-year study period, 5941 respiratory samples from patients with RTIs (females: 3101, 52.2 % and males: 2840, 47.8 %) were analysed for the presence of HBoV using multiplex real-time PCR. The age of the patients ranged from 1 month to 106 years, with a median age of 31 years. Among these patients, 2332 (39.3 %) were Kuwaiti and 3609 (60.7 %) were non-Kuwaiti (*P*<0.0001). HBoV was detected in 111/5941 (1.9 %) samples. Among the HBoV-positive patients, 59 (53.2 %) were males and 52 (46.8 %) were females (*P*=0.5029), with a median age of 1 year. The low rate of HBoV infection in this study is in parallel with another study in PR China, in which HBoV was detected in 1.6 % of respiratory samples from children with acute respiratory infections [19]. Schildgen *et al*. [20] reported a similarly low rate. Conversely, others reported higher HBoV prevalence: 4.9 % in Kuwait was reported by Essa *et al*. [21], 7.2–33 % by Tran *et al*. [22], 10.3 % by Weissbrich *et al*. [23], 12.2 % by Ahn *et al*. [24], and 33 % by Martin *et al*. [25]. Differences in sample collection, differences in the study group involved (young, adult or elder), and regional and temporal differences in other studies may account for variations in positive rates.

**Fig. 1.**
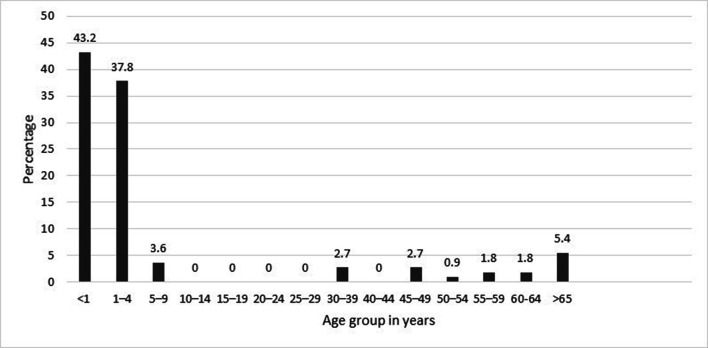
Distribution of HBoV-positive patients by age.

The age distribution of HBoV-positive patients is shown in Fig. 1. The results revealed that the prevalence of HBoV was high in children younger than 5 years of age, with peak prevalence occurring in children <1 year old (42.3 %), while none of the patients aged 10–29 years old were infected with HBoV. These results are in close agreement with many studies that have shown that infants and children <5 years old have the highest rate of HBoV infection [17, 26, 27]. Our results suggested that children <5 years old are more susceptible to HBoV infection due to the waning of maternally acquired antibodies. However, the lower prevalence of HBoV in older children and adults, as shown in other studies [14, 28, 29], may be due to immunity acquired from previous infection in early childhood.

**Fig. 2.**
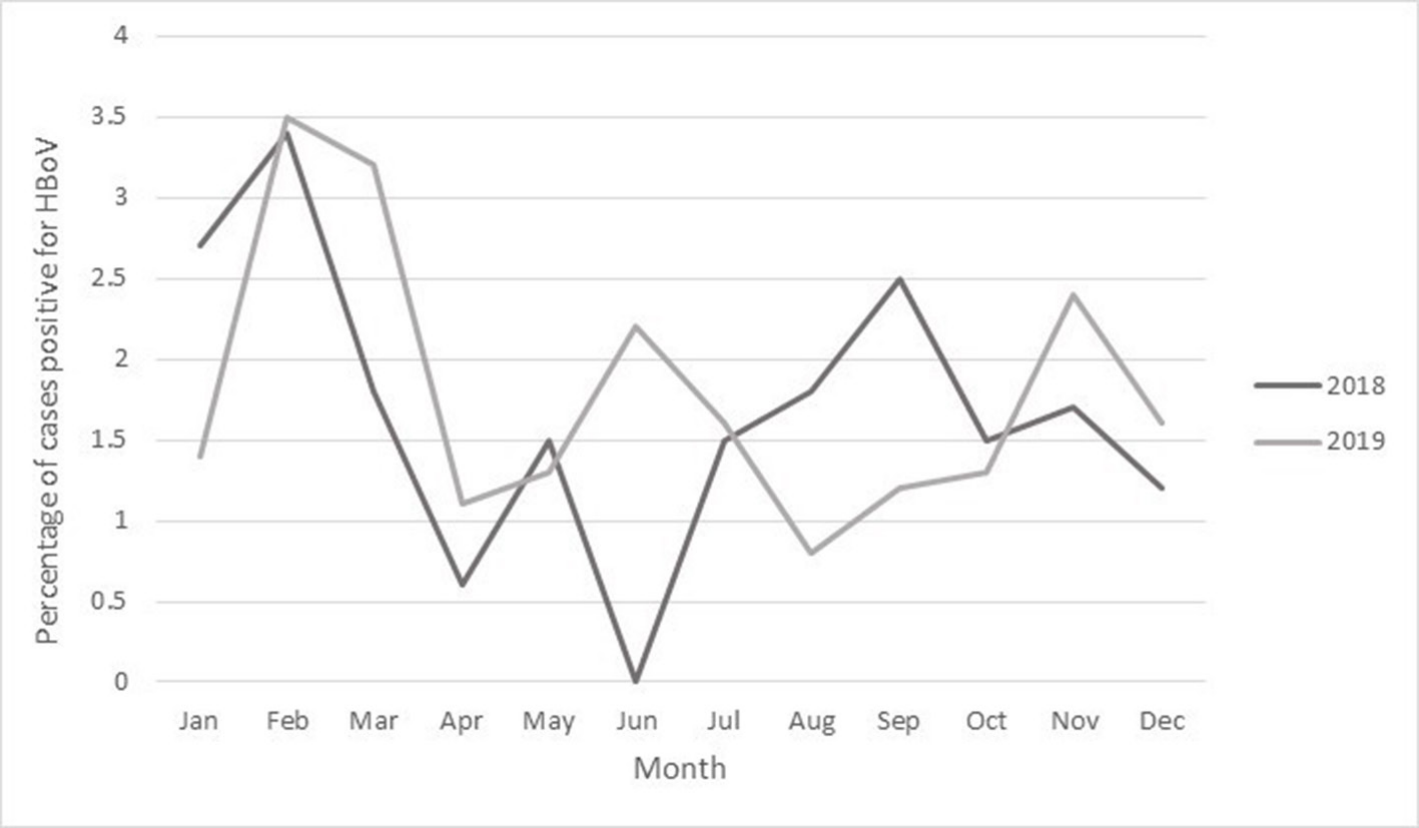
Monthly distribution of HBoV-positive cases from January 2018 to January 2020.

Seasonal distribution of HBoV was noted throughout the year (from January to December), with the peak occurring in February in both 2018 and 2019 (Fig. 2). In June 2018, HBoV infection was not detected among patients. These findings are in agreement with the findings of a study by Silva *et al*. [30], who showed that the occurrence of HBoV was more frequently associated with autumn and spring, while others showed a higher prevalence of the virus in winter [1] or summer [24]. However, another study did not observe any seasonal preference of the virus in patients with respiratory RTIs [19]. Thus, the seasonal preference of HBoV infections varies due to variation in climates and various factors affecting the prevalence of respiratory infections in each country.

Among the 111 patients who were positive for HBoV, HBoV alone was detected in 48 (43.2 %), while the remaining 63 (56.8 %) had co-infections with other viruses, without significant differences between the two types of infection being observed (*P*=0.1575) (Table 1). In the category of co-infection, the most common co-infection was with RSV (10.8 %), followed by HRV (9.9 %) and influenza A virus (IAV) (6.3 %) (Table 1). Co-infection with more than one viral agent was found in 15 % of the patients. The detection of HBoV co-infection with other viruses in patients with respiratory diseases has been demonstrated in many studies. In these studies, the rate of HBoV co-infection ranged from 17.6–84.4 % [31–34]. The study by Sloots *et al*. [35] showed comparable results; they detected HBoV co-infection with other viruses in 55.6 % of patients suffering from ARTI, most frequently HRSV. Besides, global analysis has revealed various frequencies of HBoV co-infection with HRV, RSV, influenza A/B and coronaviruses in patients with respiratory diseases [8]. Indeed, HBoV has been regarded as an infectious agent; however, its pathogenic role in respiratory disease is still arguable. This virus is frequently detected in co-infections with other respiratory viruses with a well-established pathogenic role, but HBoV is still rare in asymptomatic individuals [36]. There are many hypotheses for this; one is that shedding of HBoV is enhanced by respiratory tract inflammation caused by another virus. Another hypothesis is that HBoV maybe a helper virus that aids other viruses or itself requires the presence of other viruses for its activation or reactivation [8].

**Table 1.** Distribution of HBoV single infection and co-infection with other viruses

Type of infection	Virus					No. of patients (%)	Subtotal (%)
Single infection	HBoV (only)					48 (43.2)	48 (43.2)
Co-infection	HBoV	RSV				12 (10.8)	63 (56.8)
	HBoV	HRV				11 (9.9)	
	HBoV	IAV				7 (6.3)	
	HBoV	ADV				4 (3.6)	
	HBoV	HCoV (OC43)				3 (2.7)	
	HBoV	HMPV				3 (2.7)	
	HBoV	HRV	RSV			2 (1.8)	
	HBoV	HDV	HRV			2 (0.8)	
	HBoV	PIV3				1 (0.9)	
	HBoV	PIV3	ENT			1 (0.9)	
	HBoV	PIV3	RSV			1 (0.9)	
	HBoV	HCoV (OC43)	HSV1			1 (0.9)	
	HBoV	HCoV (64)				1 (0.9)	
	HBoV	HCoV (OC43)	RSV			1(0.9)	
	HBoV	HCoV (OC43)	ADV	ENT		1 (0.9)	
	HBoV	IBV				1 (0.9)	
	HBoV	HCoV (NL63)				1 (0.9)	
	HBoV	HCoV (64)	IAV			1 (0.9)	
	HBoV	ENT				1 (0.9)	
	HBoV	IAV (H1N1)				1 (0.9)	
	HBoV	IBV	RSV			1 (0.9)	
	HBoV	HPeV	RSV			1 (0.9)	
	HBoV	HRV	HCoV (OC43)	PIV3	RSV	1 (0.9)	
	HBoV	HRV	ENT			1 (0.9)	
	HBoV	HRV	IAV	RSV		1 (0.9)	
	HBoV	HRV	PIV3	HPeV		1 (0.9)	
	HBoV	HCoV (OC43)	HRV			1 (0.9)	
**Total**							111 (100)

HBoV, human bocavirus; HRV, human rhinovirus; RSV, respiratory syncytial virus; IAV, influenza A virus; ADV, adenovirus; PIV, parainfluenza virus; ENT, enterovirus; HCoV, human coronavirus; HMPV, human metapneumovirus; HSV, human simplex virus; IBV, influenza virus B virus; HPeV, parechovirus.

The clinical characteristics of HBoV-positive patients are summarized in Table 2. The most predominant symptom among both single and co-infection cases was fever (70.8 and 55.6 %, respectively), followed by cough (69.8 and 53.9 %, respectively). Respiratory distress was the third most common symptom detected in both single and co-infection cases (35.4 and 41.3 %, respectively). Rhinorrhea was significantly more common in single infection than in co-infection cases (33.3 vs 17.5 %; *P*=0.05). On the other hand, respiratory failure was only detected in single HBoV infection cases (6.3 % of patients) (*P*=0.04). Wheezing was detected more frequently in co-infection than single infection cases, with a noteworthy difference (15.9 vs 2.1 %; *P*=0.01). Although pneumonia was more frequently associated with co-infection (11.1 %) than single infection (4.2 %) cases, a substantial difference was not detected (*P*=0.18). In agreement with our study, other studies have shown that the most common symptoms detected in patients who are solely positive for HBoV are cough, rhinorrhea and fever. Further, HBoV-infected patients in cases of single infection or co-infection with other viruses have a similar clinical picture to those seen in patients infected with other respiratory viruses, such as RSV and HMPV, with symptoms including upper RTI, bronchitis, bronchiolitis, pneumonia and acute exacerbation of asthma [37, 38]. As with our results, Deng *et al*. [39] and Fry *et al*. [17] showed that wheezing was detected significantly more frequently in patients with co-infection compared to single HBoV infection.

**Table 2.** Comparison of clinical symptoms between HBoV single infection and co-infection with other viruses

Clinical symptoms	Single infection (%), *n*=48	Co-infection (%), *n*=63	*P* value
**Fever**	70.8	55.6	0.10
**Cough**	69.8	53.9	0.09
**Acute respiratory distress syndrome**	35.4	41.3	0.52
**Rhinorrhea**	33.3	17.5	0.05
**Shortness of breath**	18.8	9.5	0.15
**Respiratory failure**	6.3	0	0.04
**Pneumonia**	4.2	11.1	0.18
**Seizure**	4.2	3.2	0.78
**Wheeze**	2.1	15.9	0.01
**Acute otitis media**	2.1	0	0.25
**Headache**	2.1	0	0.25
**Bronchopneumonia**	0	1.6	0.38

**Fig. 3.**
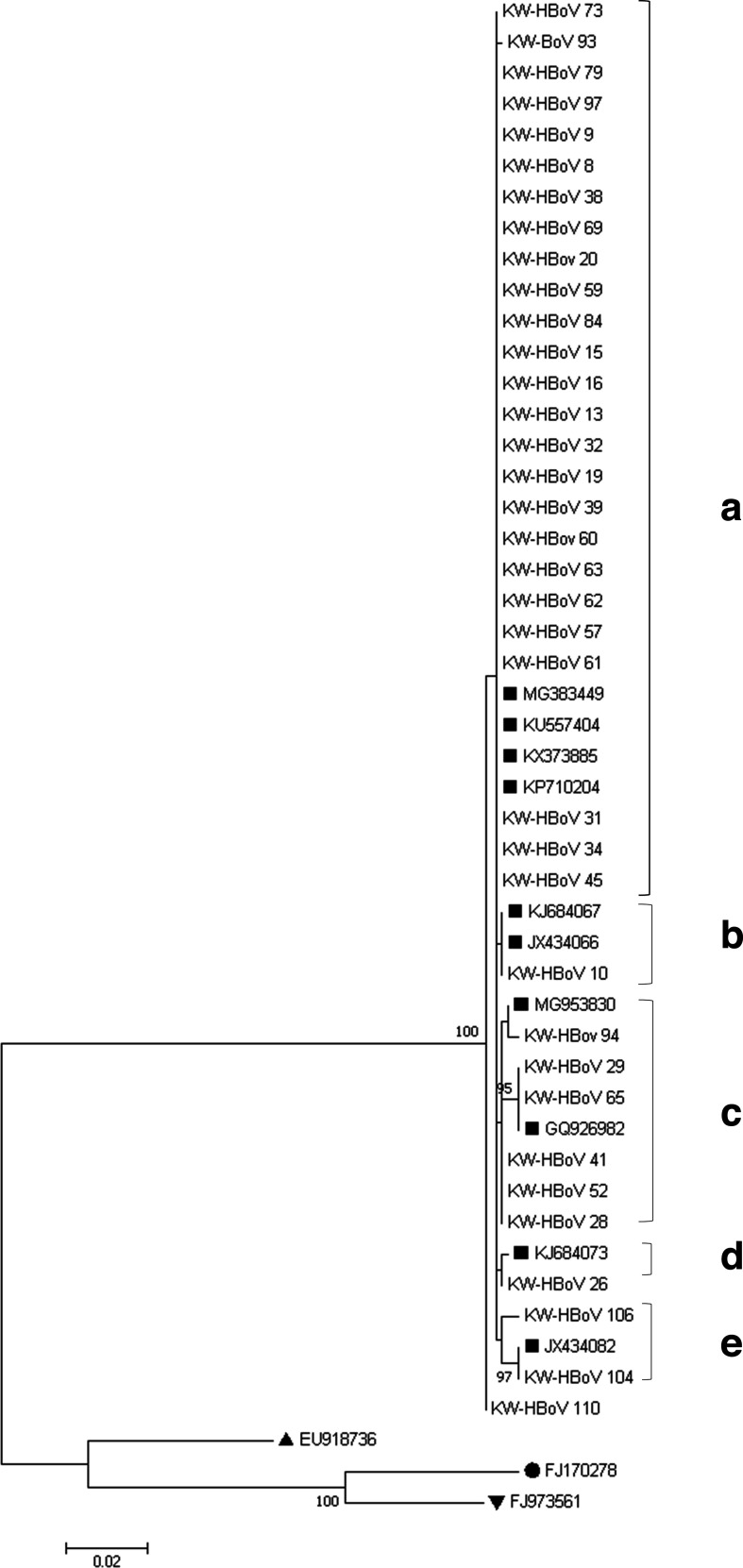
Phylogenetic analysis of partial sequences of the NP1 and VP1/VP2 genes (≈748) of 36 HBoV-1 isolates from patients. The topology tree was constructed using the maximum-likelihood method and mega 7.0. The stability of the tree was evaluated by using 1000 bootstrap replicates. Only bootstrap values greater than 70 % are shown on the branch. ■, HBoV-1 sequence from GenBank; ●, HBoV-2 sequence from GenBank; ▲, HBoV-3 sequence from GenBank and ▼, HBoV-4 sequence from GenBank.

Nucleotide sequencing of partial NP1 and VP1/VP2 regions (≈748 bp) was performed successfully for 36/111 HBoV strains isolated from patients with RTIs in Kuwait. This decreased amplification rate is due to the increased sensitivity of the multiplex real-time PCR compared to the in-house PCR assay for genotyping or due to low viral load in some samples. Sequence analysis of the partial NP1, VP1/VP2 genes obtained from the HBoV-positive patients revealed that all 36 Kuwaiti strains belonged to the HBoV-1 genotype, with HBoV-2, HBoV-3 and HBoV-4 not being detected (Fig. 3). These results are in agreement with other studies, which have shown that HBoV-1 is a respiratory pathogen and is associated with respiratory illness, while HBoV-2, 3 and 4 are not directly involved in respiratory tract infections [15, 24, 40]. On the other hand, some reports have described an HBoV2–4 prevalence of 0.4–4.3 % in respiratory samples, but their roles in respiratory diseases are uncertain [41, 42].

Phylogenetic analysis demonstrated that the Kuwaiti HBoV-1 strains are highly conserved with a low degree of genetic variability. Similar results were revealed in previous studies [24, 43–46]. Our results demonstrated that the Kuwaiti HBoV-1 strains were clustered into five five main lineages (A–E) with high nucleotide identity (98.8–100 %) between all Kuwaiti HBoV sequences. Lineage A contained 25/36 (96 %) identical Kuwaiti HBoV strains (MT370511, LR721646, MT370510, MT409211) that clustered with the MG383449 Ethiopian strain, KU557404 Egyptian strain, KX373885 Mexican strain and KP710204 Chinese strain with 100 % identity. Lineage B comprised one Kuwaiti HBoV strain that clustered with the JX43066 and KJ684067 Chinese strains with 100 % identity at the nucleotide level. Lineage C, on the other hand, had three sublineages; the first one contained the Kuwaiti strain HBoV94 that showed 99.7 % sequence identity with the MG953830 Brazilian strain at the nucleotide level, whereas the second sublineage contained two Kuwaiti strains (MT409214, MT409215) that were 100 % identical at the nucleotide level with the GQ926982 Brazilian strain. The third sublineage of cluster C comprised three identical Kuwaiti strains (MT409216, MT409217, MT409218) sharing 99.8 % identity with the MG953830 Brazilian strain at the nucleotide level. Lineage D contained only one Kuwaiti strain (MT409219) that showed 99.8 % identity at the nucleotide level with the KJ684073 Chinese strain. The last lineage (E) contained the Kuwaiti HBoV104 (MT409220) that showed 100 % identity at the nucleotide level with the JX434082 Chinese strain. However, the Kuwaiti strain HBoV110 (MT409221) clustered with the MG383449 Ethiopian strain with 99.6 % identity at the nucleotide level. As most of the nucleotide variations were conservative at the amino acid level, there is 0–1.1% divergence among all Kuwaiti strains. Most of the nucleotide variability occurred in the VP1/VP2 genes; however, the Kuwaiti strain HBoV104 showed extra variability in three nucleotide positions in the NP1 gene. The analysis demonstrated that 18 out of 748 nucleotide positions were found to be variable, with 12 (66.7 %) being singleton sites, while six (33.3 %) positions were parsimony-informative sites. In parallel to our study, Allander *et al*. [47] showed that nucleotide polymorphisms were most common in the VP1/VP2 gene of HBoV. Based on our findings, we speculate that because of the low level of genetic variability in HBoV strains among patients with RTIs in Kuwait, the resulting immunity is life-long and recurrent infections with HBoV will be rare.

In conclusion, this study provided data on the epidemiology of HBoV among patients with RTIs in Kuwait. The data showed that HBoV-1 was the dominant circulating genotype in Kuwait. Furthermore, patients infected with HBoV exhibited several respiratory symptoms as a result of single or co-infections, confirming its role in respiratory diseases. Phylogenetic analysis also showed that the circulating strains of HBoV-1 in Kuwait exhibited low genetic variability and more than half of the strains belonged to a single lineage of HBoV-1.

## References

[1] Allander T, Tammi MT, Eriksson M, Bjerkner A, Tiveljung-Lindell A (2005). From the cover: cloning of a human parvovirus by molecular screening of respiratory tract samples. Proc Natl Acad Sci.

[2] Lekana-Douki SE, Behillil S, Enouf V, Leroy EM, Berthet N (2018). Detection of human bocavirus-1 in both nasal and stool specimens from children under 5 years old with influenza-like illnesses or diarrhea in Gabon. BMC Res Notes.

[3] Chen Y, Liang W, Yang S, Wu N, Gao H (2013). Human infections with the emerging avian influenza A H7N9 virus from wet market poultry: clinical analysis and characterization of viral genome. Lancet.

[4] Chen AY, Cheng F, Lou S, Luo Y, Liu Z (2010). Characterization of the gene expression profile of human bocavirus. Virology.

[5] Kapoor A, Simmonds P, Slikas E, Li L, Bodhidatta L (2010). Human bocaviruses are highly diverse, dispersed, recombination prone, and prevalent in enteric infections. J Infect Dis.

[6] Xu Z-qian, Cheng W-xia, Li B-wen, Li J, Lan B (2011). Development of a real-time PCR assay for detecting and quantifying human bocavirus 2. J Clin Microbiol.

[7] Moffatt S, Yaegashi N, Tada K, Tanaka N, Sugamura K Human parvovirus B19 nonstructural (NS1) protein induces apoptosis in erythroid lineage cells. J Virol.

[8] Schildgen O, Müller A, Allander T, Mackay IM, Völz S (2008). Human bocavirus: passenger or pathogen in acute respiratory tract infections?. Clin Microbiol Rev.

[9] McIntosh K (2006). Human bocavirus: developing evidence for pathogenicity. J Infect Dis.

[10] Lindner J, Modrow S (2008). Human bocavirus--a novel parvovirus to infect humans. Intervirology.

[11] Chieochansin T, Chutinimitkul S, Payungporn S, Hiranras T, Samransamruajkit R (2007). Complete coding sequences and phylogenetic analysis of human bocavirus (HBoV). Virus Res.

[12] Blinkova O, Rosario K, Li L, Kapoor A, Slikas B (2009). Frequent detection of highly diverse variants of Cardiovirus, cosavirus, bocavirus, and circovirus in sewage samples collected in the United States. J Clin Microbiol.

[13] Guido M, Tumolo MR, Verri T, Romano A, Serio F (2016). Human bocavirus: current knowledge and future challenges. World J Gastroenterol.

[14] Bastien N, Brandt K, Dust K, Ward D, Li Y (2006). Human bocavirus infection, Canada. Emerg Infect Dis.

[15] Jartti T, Hedman K (2012). Human bocavirus-the first 5 years. Rev Med.

[16] Fredricks DN, Relman DA (1996). Sequence-Based identification of microbial pathogens: a reconsideration of Koch's postulates. Clin Microbiol Rev.

[17] Fry AM, Lu X, Chittaganpitch M, Peret T, Fischer J (2007). Human bocavirus: a novel parvovirus epidemiologically associated with pneumonia requiring hospitalization in Thailand. J Infect Dis.

[18] Kumar S, Stecher G, Tamura K (2016). MEGA7: molecular evolutionary genetics analysis version 7.0 for bigger datasets. Mol Biol Evol.

[19] Zhao M, Zhu R, Qian Y, Deng J, Wang F (2014). Prevalence analysis of different human bocavirus genotypes in pediatric patients revealed intra-genotype recombination. Infect Genet Evol.

[20] Schildgen O, Müller A, Allander T, Mackay IM, Völz S (2008). Human bocavirus: passenger or pathogen in acute respiratory tract infections?. Clin Microbiol Rev.

[21] Essa S, Owayed A, Altawalah H, Khadadah M, Behbehani N (2015). The prevalence of human bocavirus, human coronavirus-NL63, human metapneumovirus, human polyomavirus KI and Wu in respiratory tract infections in Kuwait. Med Princ Pract.

[22] Tran DN, Nguyen TQN, Nguyen TA, Hayakawa S, Mizuguchi M (2014). Human bocavirus in children with acute respiratory infections in Vietnam. J Med Virol.

[23] Weissbrich B, Neske F, Schubert J, Tollmann F, Blath K (2006). Frequent detection of bocavirus DNA in German children with respiratory tract infections. BMC Infect Dis.

[24] Ahn JG, Choi SY, Kim DS, Kim KH (2014). Human bocavirus isolated from children with acute respiratory tract infections in Korea, 2010-2011. J Med Virol.

[25] Martin ET, Fairchok MP, Kuypers J, Magaret A, Zerr DM (2010). Frequent and prolonged shedding of bocavirus in young children attending daycare. J Infect Dis.

[26] Kesebir D, Vazquez M, Weibel C, Shapiro ED, Ferguson D (2006). Human bocavirus infection in young children in the United States: molecular epidemiological profile and clinical characteristics of a newly emerging respiratory virus. J Infect Dis.

[27] Maggi F, Andreoli E, Pifferi M, Meschi S, Rocchi J (2007). Human bocavirus in Italian patients with respiratory diseases. J Clin Virol.

[28] Noh JY, Song JY, Cheong HJ, Choi WS, Lee J (2013). Laboratory surveillance of influenza-like illness in seven teaching hospitals, South Korea: 2011-2012 season. PLoS One.

[29] Manning A, Russell V, Eastick K, Leadbetter GH, Hallam N (2006). Epidemiological profile and clinical associations of human bocavirus and other human parvoviruses. J Infect Dis.

[30] Silva PE, Figueiredo CA, Luchs A, de Paiva TM, Pinho MAB (2018). Human bocavirus in hospitalized children under 5 years with acute respiratory infection, São Paulo, Brazil. Arch Virol.

[31] Arden KE, McErlean P, Nissen MD, Sloots TP, Mackay IM (2006). Frequent detection of human rhinoviruses, paramyxoviruses, coronaviruses, and bocavirus during acute respiratory tract infections. J Med Virol.

[32] Naghipour M, Cuevas LE, Bakhshinejad T, Dove W, Hart CA (2007). Human bocavirus in Iranian children with acute respiratory infections. J Med Virol.

[33] Ljubin-Sternak S, Meštrović T, Ivković-Jureković I, Tešović G, Mlinarić-Galinović G, Lukšić I (2019). High detection rates of human bocavirus in infants and small children with lower respiratory tract infection from Croatia. Clin Lab.

[34] Lekana-Douki SE, Behillil S, Enouf V, Leroy EM, Berthet N (2018). Detection of human bocavirus-1 in both nasal and stool specimens from children under 5 years old with influenza-like illnesses or diarrhea in Gabon. BMC Res Notes.

[35] Sloots TP, McErlean P, Speicher DJ, Arden KE, Nissen MD (2006). Evidence of human coronavirus HKU1 and human bocavirus in Australian children. J Clin Virol.

[36] Chow BDW, Esper FP (2009). The human bocaviruses: a review and discussion of their role in infection. Clin Lab Med.

[37] Weigl JAI, Puppe W, Schmitt H-J (2004). Variables explaining the duration of hospitalization in children under two years of age admitted with acute airway infections: does respiratory syncytial virus have a direct impact?. Klin Padiatr.

[38] Wilkesmann A, Schildgen O, Eis-Hübinger AM, Geikowski T, Glatzel T (2006). Human metapneumovirus infections cause similar symptoms and clinical severity as respiratory syncytial virus infections. Eur J Pediatr.

[39] Deng Y, Gu X, Zhao X, Luo J, Luo Z (2012). High viral load of human bocavirus correlates with duration of wheezing in children with severe lower respiratory tract infection. PLoS One.

[40] Kenmoe S, Vernet M-A, Njankouo-Ripa M, Penlap VB, Vabret A (2017). Phylogenic analysis of human bocavirus detected in children with acute respiratory infection in Yaounde, Cameroon. BMC Res Notes.

[41] TaeHee H, JuYoung C, EungSoo H Human bocavirus 2 in children, South Korea. Emerg Infect Dis.

[42] Koseki N, Teramoto S, Kaiho M, Gomi-Endo R, Yoshioka M (2012). Detection of human bocaviruses 1 to 4 from nasopharyngeal swab samples collected from patients with respiratory tract infections. J Clin Microbiol.

[43] Abdel-Moneim AS, Kamel MM, Hassan NM (2017). Evolutionary and genetic analysis of human bocavirus genotype-1 strains reveals an evidence of intragenomic recombination. J Med Microbiol.

[44] Allander T, Jartti T, Gupta S, Niesters HGM, Lehtinen P (2007). Human bocavirus and acute wheezing in children. Clin Infect Dis.

[45] Neske F, Blessing K, Tollmann F, Schubert J, Rethwilm A (2007). Real-Time PCR for diagnosis of human bocavirus infections and phylogenetic analysis. J Clin Microbiol.

[46] Lee EJ, Kim H-S, Kim HS, Kim J-S, Song W (2016). Human bocavirus in Korean children with gastroenteritis and respiratory tract infections. Biomed Res Int.

[47] Allander T, Tammi MT, Eriksson M, Bjerkner A, Tiveljung-Lindell A (2005). Cloning of a human parvovirus by molecular screening of respiratory tract samples. Proc Natl Acad Sci U S A.

